# The Emerging Roles of JNK Signaling in *Drosophila* Stem Cell Homeostasis

**DOI:** 10.3390/ijms22115519

**Published:** 2021-05-24

**Authors:** Salvador C. Herrera, Erika A. Bach

**Affiliations:** 1 Centro Andaluz de Biología del Desarrollo, CSIC/Universidad Pablo de Olavide/JA, Carretera de Utrera km 1, 41018 Sevilla, Spain; 2 Department of Biochemistry and Molecular Pharmacology, New York University Grossman School of Medicine, New York, NY 10016, USA; 3 Helen L. and Martin S. Kimmel Center for Stem Cell Biology, New York University Grossman School of Medicine, New York, NY 10016, USA

**Keywords:** *Drosophila*, niche, germline stem cells, somatic stem cells, testis, intestine, intestinal stem cells, enterocytes, proliferation, cell death, differentiation, inflammation, regeneration, cellular plasticity, cell reprogramming

## Abstract

The Jun N-terminal kinase (JNK) pathway is an evolutionary conserved kinase cascade best known for its roles during stress-induced apoptosis and tumor progression. Recent findings, however, have identified new roles for this pleiotropic pathway in stem cells during regenerative responses and in cellular plasticity. Here, we provide an overview of recent findings about the new roles of JNK signaling in stem cell biology using two well-established *Drosophila* models: the testis and the intestine. We highlight the pathway’s roles in processes such as proliferation, death, self-renewal and reprogramming, and discuss the known parallels between flies and mammals.

## 1. Introduction

Stem cells are essential for the maintenance of many adult tissues, and they support tissues as a result of two key properties: (1) their capacity for self-renewal, by which they can proliferate while maintaining their stemness, and (2) their ability to produce differentiating daughter cells, which replace effete cells in tissues with a high cellular turnover. Tissue homeostasis requires the precise regulation of the balance between self-renewal and differentiation of these cells. Moreover, stem cells exhibit plasticity to cope with the stresses, insults and injuries that animals experience during their life. This plasticity allows stem cells to modify their activity, enabling tissue repair and modulating tissue function. Frequently, stem cells reside in “niches”, specialized micro-environments that regulate both self-renewal and differentiation and that are responsive to environmental and physiological alterations.

JNK is a conserved branch of the mitogen-activated protein kinase (MAPK) pathway (reviewed in [1,2]). The core JNK pathway in mammals is a very complex kinase cascade composed by two different JNK kinase kinases (MKK4 and MKK7) phosphorylating three alternative JNKs (JNK1, JNK2 and JNK3) that can be alternatively spliced into more than 10 isoforms. Downstream of JNK phosphorylation, the AP-1 transcription factor complex can be formed from two Jun (Jun, JunD) and three Fos (Fos, FosL1, FosL2) alternative proteins. By contrast, in *Drosophila*, the pathway is genetically much simpler, making flies a convenient model for its study.

**Figure 1:**
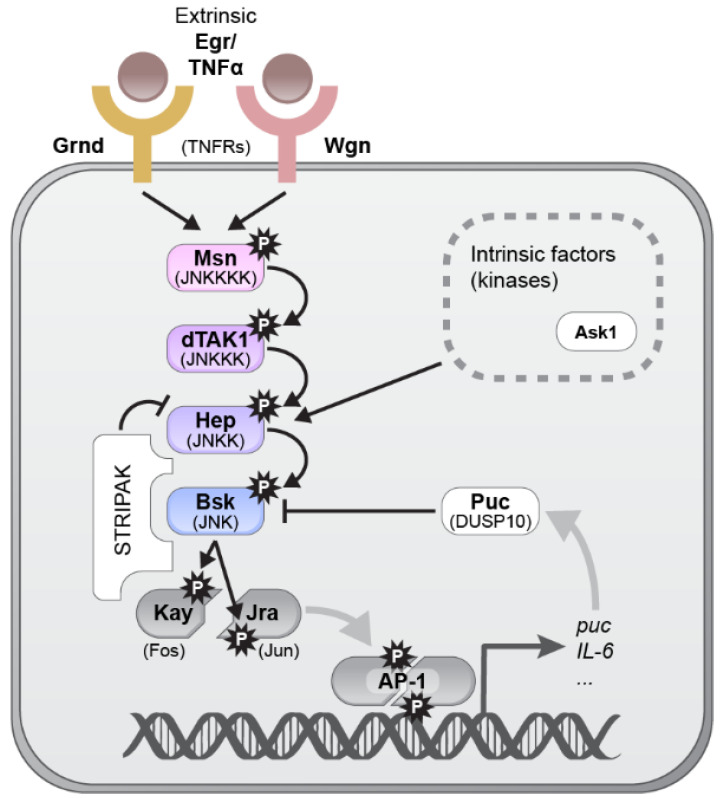
The JNK pathway in *Drosophila*. Extrinsic signals such as the ligand Egr/TNFα can bind the TNFα receptors (TNFRs) Grnd and Wgn, activating a cascade of kinases: Msn/JNKKKK, dTAK1/JNKKK, Hep/JNKK and Bsk/JNK. Hep/JNKK can also be phosphorylated by intrinsic factors (outlined by the dashed line), such as Ask1. Bsk/JNK phosphorylates the AP-1 transcription factor components Kay/Fos and Jra/Jun. AP-1 target genes include *puc/DUSP10*, which encodes a phosphatase that inactivates Bsk/JNK in a negative feedback loop, and *upd/IL-6*, encoding an inflammatory cytokine. The STRIPAK complex that binds to Hep/JNKK, Bsk/JNK and AP-1 and inhibits the pathway.

The *Drosophila* core JNK pathway culminates in the JNK kinase (called Hemipterous (Hep)) phosphorylating and activating one JNK (termed Basket (Bsk)) (Figure 1). The JNK pathway can be activated by the tumor necrosis factor TNF-superfamily ligand Eiger (Egr/TNFα) (reviewed in [3]), which activates two TNF receptors (TNFRs) Wengen (Wgn) [4] and Grindelwald (Grnd) [5]. Egr appears to activate JNK exclusively, while mammalian TNFα also activates NF-κB and MAPK pathways [3]. Downstream of TNFRs, Bsk/JNK phosphorylates AP-1 components Jun and Fos, which are represented by single orthologs in *Drosophila*, Jun-related antigen (Jra) and Kayak (Kay), respectively, as well as other substrates (reviewed in [6]). AP-1 transcriptionally upregulates numerous target genes. One well-established target gene is *puckered* (*puc*) (ortholog of *dual-specificity phosphatase 10* (*DUSP10*) [7], encoding a phosphatase that dephosphorylates Bsk/JNK in an induced-negative feedback loop. Other targets include *unpaired* (*upd*), encoding the *Drosophila* interleukin-6 (IL-6) homolog, and *Matrix metalloprotease 1* (*Mmp1*), encoding a protease that degrades the extracellular matrix (reviewed in [8]). In addition to Puc, JNK signaling is downregulated by the STRIPAK complex components Striatin interacting protein (Strip) and Connector of kinase to AP-1 (Cka), which bind to and inactivate Hep/JNKK [9,10,11]. JNK signaling can also be activated intrinsically, for example, through the redox-sensitive MAPKKK Apoptotic signal-regulating kinase 1 (Ask1) [12].

In both *Drosophila* and higher organisms, JNK has pleiotropic effects, including apoptosis, proliferation, differentiation, cell migration, tumorigenesis, and cell competition (reviewed in [1,6,13]). Additionally, activation of the JNK pathway is required for epithelial regeneration in flies [14]. In wounded epithelia, JNK is activated in the damaged cells to ensure their apoptotic death and in the nearby blastema of the surviving cells to promote their cellular reprogramming and proliferation [15,16,17,18,19,20,21,22]. This paradox of JNK inducing opposite responses in neighboring cells highlights the importance of the cellular context in triggering its diverse outcomes.

In mammalian stem cells, JNK has been studied primarily for its role in inducing apoptosis under stressful conditions [23]. However, recent work in *Drosophila* has shown that the roles of JNK in stem cells are considerably broader and include self-renewal during homeostasis, cellular plasticity during chronic stress, and pathological responses during aging. In this review, we focus on JNK function in two well-studied *Drosophila* stem cell models, the testis and the intestine, describing recent advances and relating these discoveries to relevant mammalian counterparts.

## 2. The Role of JNK in the *Drosophila* Testis during Homeostasis

**Figure 2:**
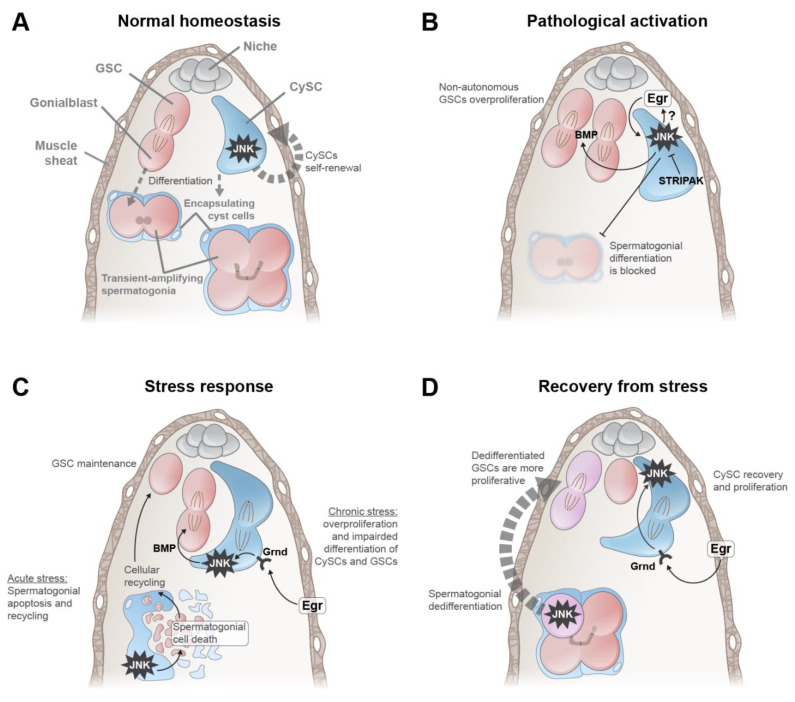
Roles of the JNK pathway in the *Drosophila* testis. (**A**) Normal homeostasis. The testis is surrounded by a sheath of smooth muscle cells (brown). Niche cells (gray) are anchored to the muscle sheath and secrete self-renewal factors for GSCs (red) and CySCs (blue). A GSC divides asymmetrically to produce a GSC daughter and a differentiating daughter (termed the Gonialblast (Gb)), which undergoes transit-amplifying divisions, producing spermatogonia. A CySC divides to produce cyst cells that become quiescent and encapsulate spermatogonia and remain attached to germ cells throughout their differentiation into spermatids. During homeostasis, JNK is intrinsically activated in CySCs and promotes their self-renewal. (**B**) Pathological activation. Impairment of the STRIPAK complex or the endocytic pathway strongly induces JNK in somatic cells, which produce abnormally high levels of Dpp/BMP. Sustained secretion of Dpp/BMP into the testis lumen causes hyper-proliferation of GSCs and early spermatogonia, while concomitantly blocking spermatogonial differentiation. These effects are genetically dependent on Egr/TNFα, which is likely produced by CySCs and then acts in an autocrine manner. (**C**) Stress response. During acute stress (left), somatic JNK activity causes the non-autonomous death of spermatogonial cells, and these somatic cells endocytose and recycle the debris of the dying germ cells. This recycling is necessary for maintaining the GSC pool during stress, presumably because recycled cellular components are somehow provided to GSCs. Under chronic stress (right), the muscle sheath secretes Egr/TNFα, which is transduced by CySCs through Grnd/TNFR. This JNK activity causes hyper-proliferation of CySCs. JNK-activated CySCs secrete Dpp/BMP, which strongly induces GSC proliferation. (**D**) Recovery from stress. Upon termination of starvation (left), JNK autonomous activation in one or more cells in a spermatogonium (pink cell with starred JNK) induces dedifferentiation into GSCs. This dedifferentiated GSC (pink dividing cells at niche) is more proliferative than the wild-type (i.e., non-dedifferentiated) sibling GSC (red cell at niche). Additionally, upon termination of starvation (right), Egr/TNFα is secreted by the muscle sheath cells and is transduced by CySCs through Grnd/TNFR. JNK-activated CySCs increase their proliferation, which facilitates the quick recovery of the CySC pool.

The *Drosophila* testis is surrounded by smooth muscle and its underlying basal lamina, similar to the seminiferous tubules of mammalian testes [24,25]. The stem cell niche is anchored to basal lamina at the apical tip of the testis (Figure 2A). The niche supports two resident stem cell populations by secreting short-range self-renew cues such as the bone morphogenetic protein (BMP) decapentaplegic (Dpp) and by providing cell–cell adhesion for germline stem cells (termed GSCs) and somatic stem cells (termed CySCs) (reviewed in [26]). GSCs divide to produce a daughter GSC that remains in contact with the niche and another daughter, the gonialblast (Gb), that differentiates after being encapsulated by somatic support cells. The Gb undergoes four transit-amplifying divisions with incomplete cytokinesis, giving rise to a 16-cell spermatogonium that enters meiosis and produces 64 individual spermatids [27]. CySCs are the functional equivalent of Sertoli cells in mammalian testes. They act as an extended niche for GSCs by secreting the self-renewal protein Dpp/BMP [28,29]. CySCs divide to produce somatic support cells, called cyst cells, that exit the cell cycle and encapsulate the Gb [30,31]. CySCs and their differentiating daughter cells, referred to as the somatic lineage, are essential for germline differentiation.

Despite intensive investigation of numerous conserved signaling networks in the *Drosophila* testis, JNK was, until recently, underexplored in this tissue. Basal levels of phosphorylated Bsk/JNK and the downstream effectors Puc and Mmp1 are detected in somatic cells of the developing and adult testis but not in GSCs or differentiating germ cells (Figure 2A and [32,33,34,35,36]). Individual CySCs homozygous mutant for *bsk*/*JNK* cannot self-renew and instead differentiate, suggesting that JNK is required for CySC niche residency under normal conditions [35]. Consistent with this mosaic analysis, somatic downregulation of Bsk/JNK reduces the number of CySCs and cyst cells [35]. However, this result was not observed by another group [34]. Nevertheless, in CySC self-renewal, JNK is activated by as-yet unidentified intrinsic factors because testes devoid of the ligand Egr/TNFα or Grnd/TNRF do not show alterations in CySC number or in CySC self-renewal [32,35].

Additional insights about the role of JNK pathway in the testis have been made by studying mutations causing ectopic JNK activity (Figure 2B). In the developing gonad, mutations in STRIPAK complex genes lead to ectopic JNK activation in somatic cells. AP-1 activation in somatic cells induces production of Egr/TNFα, which appears to act in an autocrine manner to maintain high somatic JNK activity. Ultimately, this STRIPAK/JNK/TNFα positive feedback loop disrupts somatic morphology and differentiation. These somatic perturbations have non-autonomous effects on the germline: an increased number of spermatogonia that do not differentiate beyond the transit-amplifying stage because they are improperly encapsulated by mutant somatic cells [32]. Similar phenotypes were observed in the adult testis upon mis-expression of dominant-active JNK or reduction of the endocytic pathway genes in CySCs. In both cases, the ectopic autonomous JNK activation in CySCs causes increased secretion of Dpp/BMP, which in turn increases GSC proliferation and inhibits differentiation [34,35]. Taken together, these studies reveal that pathological activation of the JNK pathway disrupts morphology, cellular behavior and transcription of somatic cells in the testis.

## 3. The Role of JNK in the *Drosophila* Testis during Stress

Additional roles of the JNK pathway have been found during episodes of stress (Figure 2C). In many organisms, nutritional deprivation causes the reversible interruption of gametogenesis. For example, in adult *C. elegans*, inadequate food intake triggers diapause, in which most of the germline dies through apoptosis. However, the adult germline can be regenerated when nutrient uptake is resumed because a small group of GSCs are protected from apoptosis during diapause [37]. In the gonads of both *Drosophila* males and females, protein deprivation (typically referred as starvation) produces a reversible tissue involution and arrest of gametogenesis [38,39]. In the testis, protein starvation reduces the number of GSCs and CySCs [33,39,40]. GSC or CySC number can be restored after five or two days, respectively, of refeeding with standard food [33,39,41]. Interestingly, the maintenance of the reduced GSC pool during starvation depends upon JNK signaling. Specifically, this reduced GSC pool is thought to be maintained by recycling cellular materials from dying transient-amplifying spermatogonia, whose death is triggered non-autonomously by JNK signaling in the encapsulating somatic support cells [40]. This process resembles the induced death of pre-meiotic spermatocytes caused by JNK-dependent loss of polarity in somatic support cells [36].

In addition to protein starvation, mating is another JNK-related source of stress in the *Drosophila* testis [33,35,41]. Prolonged mating (occurring for more than five weeks) in aged flies induces the expression of Egr/TNFα in the muscle sheath, which then accumulates in the testis lumen, and the upregulation of Grnd/TNFR in somatic support cells (Figure 2C). This increased expression of ligand and receptor causes JNK activation in CySCs, inducing excessive proliferation while concomitantly inhibiting somatic differentiation. JNK-activated CySCs also augment Dpp/BMP secretion, which induces the proliferation of early germ cells. However, germline differentiation is abrogated because of improper development of somatic support cells. Both germline and somatic starvation-related phenotypes can be abolished by the somatic downregulation of Grnd/TNFR or Hep/JNKK, or of Egr/TNFα in muscle. In sum, chronic stress reduces fertility in a process similar to an inflammatory response in the muscle sheath [35].

## 4. The Roles of JNK in Recovery from Stress and in Germline Plasticity

The JNK pathway is critical for the recovery of somatic and germline lineages after stress (Figure 2D). After starvation, JNK is activated in CySCs by muscle-produced Egr/TNFα acting on Grnd/TNFR [41]. This autonomous JNK activation in CySCs is necessary for recovery of the somatic lineage after starvation and thus for continued gametogenesis. Furthermore, JNK signaling is necessary for dedifferentiation of spermatogonia into new GSCs during the recovery from chronic stress [33]. In the *Drosophila* testis, under certain conditions, transient-amplifying spermatogonia can fragment, migrate back to the niche, and dedifferentiate into fully functional GSCs [42,43]. After the chronic stresses of starvation and mating, the GSC pool significantly declines, and spermatogenesis is greatly attenuated [33]. During the recovery period from these stresses, the GSC pool can be restored if JNK-dependent spermatogonial dedifferentiation occurs. Interestingly, these JNK-induced dedifferentiated GSCs are “fitter” than their wild-type GSC siblings in the same testis as they divide significantly more often and produce more spermatogonia. These results are intriguing because JNK-dependent cellular programming is also required for regeneration of developing *Drosophila* epithelia after damage or irradiation [18,19,20,21,44,45]. Future work will be needed to determine at which stage(s) of dedifferentiation JNK acts and what signals activate JNK in reverting spermatogonia [33].

In sum, the JNK pathway underlies cellular plasticity in the testis, enabling survival of resident stem cells during stress and inducing cell reprogramming to replenish stem cell pools once the stress is terminated. By contrast, chronic activation of the pathway can be deleterious for tissue function due to an imbalance between stem cell self-renewal and differentiation.

## 5. Making Sense of JNK Versatility in *Drosophila* and Vertebrate Testes

A recurring question in the JNK field is how the same pathway can elicit a diversity of responses. The first important consideration is the duration of the stress. For instance, acute starvation of three to six days does not induce Egf/TNFα in muscle cells; the CySC pool fully recovers after the termination of stress and homeostasis is restored. By contrast, chronic starvation of more than two weeks induces Egf/TNFα in muscle, which then leads to dysregulation of the germline and soma and reduced fertility [41]. These results indicate that acute JNK is not deleterious to tissue homeostasis [33,40,41], but that chronic pathway activation perturbs tissue function [35]. The second consideration is the level of pathway activation induced by the stress. For example, JNK-dependent proliferation at the niche (i.e., self-renewal) of CySCs is intrinsic and independent of Egf/TNFα. However, these same stem cells exhibit pathological hyper-proliferation during and after stress in response to Egf/TNFα produced by muscle [35,41]. The third consideration is the other signaling pathways concomitantly activated in each cell type and the cell-type specific expression of JNK substrates [46].

Similar context-dependent outcomes of JNK signaling are observed in the mammalian testis. In Sertoli cells, MAPKs regulate tight junction proteins that maintain the blood–testis barrier (BTB), which isolates germ cells during spermatogenesis [47]. However, TNFα-induced JNK activation in Sertoli cells disrupts the BTB, which then causes the non-autonomous loss of germ cells [48]. However, JNK activation in Sertoli cells is not always detrimental: after treatment with the BTB-damaging agent cadmium chloride, JNK activation protects the BTB from excessive remodeling and reduces subsequent germ cell loss [49]. In the germline, MAPKs perform several functions, including proliferation of spermatogonial stem cells (SSCs) (i.e., mammalian GSCs), meiotic entry of spermatocytes and sperm motility (reviewed in [47,50]). Recent work has shown that reactive oxygen species (ROS) activate JNK and the MAPK p38 and promote SSC self-renewal. When ROS, JNK or p38 activity is inhibited pharmacologically, SSC proliferation is reduced, as is the number of SSCs. [51]. These results are consistent with the independent observation that SSC proliferation ex vivo depends on JNK pathway activity [52]. ROS-JNK dependent proliferation in SSCs is similar to what has been reported for surviving cells during epithelial regeneration in *Drosophila* [15]. The relationship between ROS and JNK has not yet been examined in the *Drosophila* testis, but this should be addressed in the future.

## 6. JNK Signaling in Stress Responses and Aging in the *Drosophila* Intestine

**Figure 3:**
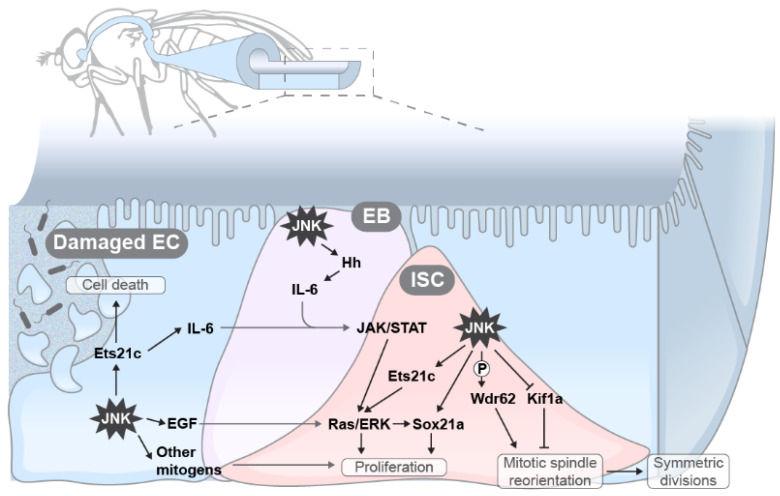
The JNK pathway during homeostasis of the *Drosophila* gut. Damage or bacterial infection in the *Drosophila* midgut causes JNK activation in enterocytes (ECs). JNK induces expression of Ets21c, which then induces the secretion of inflammatory cues and mitogens such as IL-6 and EGFs. In cases of severe or chronic damage, JNK/Ets21c activity also induces apoptosis of the EC. Enteroblasts (EBs) activate JNK upon stress. This leads to the production of Hh, which acts in an autocrine manner to induce IL-6 production in EBs. In intestinal stem cells (ISCs), IL-6 and EGFs are received and integrated by the JAK/STAT and Ras/ERK pathways, respectively, to induce stem cell proliferation. Autonomous JNK pathway activation in ISCs upregulates its effectors Ets21c and Sox21a, which promote proliferation. In addition, JNK signaling induces symmetric divisions in ISCs by reorientating the mitotic spindle through its effector Wdr62 and by transcriptionally inhibiting Kif1a.

Approximately 9 mm in length, the *Drosophila* gastrointestinal tract is divided into three regions—foregut, midgut and hindgut—which are functionally and morphologically analogous to the mammalian esophagus, small intestine and large intestine, respectively [53]. As with the small intestine, the *Drosophila* midgut epithelium is maintained by intestinal stem cells (ISCs) and has the capacity to regenerate after damage or infection (Figure 3). Under homeostatic conditions, ISCs divide infrequently and asymmetrically to produce an ISC and an enteroblast (EB). EBs do not divide again and terminally differentiate into absorptive enterocytes (ECs). The basal division rate of ISCs allows the epithelium to turnover every two weeks. Due to functional, structural and cellular similarities to the mammalian intestine, the *Drosophila* digestive tract has become a useful model for studying intestinal homeostasis, aging and disease [54,55,56,57].

ISC self-renewal and proliferation is controlled by epidermal growth factor receptor (EGFR) and other signaling pathways, whereas ISC differentiation is regulated by Notch and JAK/STAT (reviewed in [54,58,59]). After injury or bacterial infection, JNK is essential for intestinal regeneration. During stress, the JNK pathway is activated in ISCs, EBs, and ECs but serves distinct roles in each cell type (Figure 3). In ECs, activated JNK induces the expression of Ets21c, an ETS-domain transcription factor [60], and Ets21c ensures caspase-dependent death of the damaged cells and induces secretion of growth and inflammatory factors such as EGFs and IL-6 [54,61,62,63]. In EBs, JNK signaling induces IL-6 secretion, but in this cell type IL-6 expression is controlled by the Hh pathway downstream of JNK activation [64]. ISCs receive EGF and IL-6 signals produced by damaged ECs and EBs. These factors activate Ras/MAPK and JAK/STAT pathways, respectively, which promotes ISC proliferation (reviewed in [54,58]). Additionally, JNK is activated in ISCs upon damage to the midgut, but the factors activating JNK in ISCs are not yet known. In ISCs, JNK induces the expression of downstream effectors Ets21c and Sox21a, a Sox transcription factor member [60,65]. Autonomous JNK activation in ISCs synergizes with self-renewal pathways such as Ras/ERK to augment their proliferation (Figure 3) [66,67,68]. JNK can become activated in ISCs and induce ISC proliferation, even in the absence of EC death, by feeding animals with small amounts of opportunistic bacteria [68].

Environmental cues can cause ISCs to switch from homeostatic asymmetric stem cell division (in which one daughter cell retains stemness and the other differentiates) to symmetric division (in which both daughter cells retain stemness) [69]. This involves changing the orientation of ISC mitotic spindle relative to the plane of the epithelium from an oblique angle (asymmetric) to parallel (symmetric) [70]. In *Drosophila* ISCs, JNK induces symmetric cell divisions by phosphorylating and recruiting Wdr62 to the centrosome and by repressing transcription of Kif1a, a kinesin implicated in promoting asymmetric divisions [71,72]. This acute switch to ISC symmetric divisions is required for intestinal growth in young adults [69]. However, when symmetric divisions become chronic, for example, during stress or aging, tissue homeostasis is compromised and the lifespan is shortened (see below). While spindle reorientation favoring symmetric divisions has been observed in mouse ISCs, it is not known whether JNK regulates this process [70,73].

The midgut of old flies is characterized by age-related dysplasia that shortens the lifespan, and JNK signaling plays a pivotal role in this process (reviewed in [55,74]). As flies age, alterations in cell fate of middle midgut cells cause a reduction in acidification of the midgut lumen, and this causes dysbiosis of commensal microbiota [75,76]. The epithelium of the midgut then induces a chronic inflammatory response through the activation of p38 and the ROS-producing enzyme Duox. The production of ROS triggers JNK activation in ISCs, which then proliferate uncontrollably [63,67,77]. Age-related dysplasia can be prevented and the lifespan can be extended by globally abrogating the JNK pathway in *hep/JNKK* mutant flies or by downregulating *bsk/JNK* in ISCs/EBs [63,78]. Interestingly, the sustained proliferation of ISCs is driven by JNK-dependent symmetric divisions induced by the mitotic spindle reorientation described above. Downregulating *bsk/JNK* in ISCs, decreasing Wdr62 expression, or upregulating Kif1a restores the normal oblique mitotic spindles and promotes asymmetric divisions, thus reducing the number of ISCs in the epithelium and extending lifespan [71].

The JNK pathway can induce intestinal dysplasia by a different mechanism that results in multi-layered tissue architecture. In this model, damaged ECs devoid of an integrin subunit activate JNK, which induces IL-6 secretion. ISCs increase proliferation in response to IL-6 and their EB daughters differentiate into new ECs. This cycle is repeated, resulting in tissue overgrowth [79]. The JNK/IL-6 cascade is reutilized in other ISC tumor models. For example, high bacterial load can induce the JNK/IL-6 circuit [67] and, together with the expression of proto-oncogenes such as dominant-active Ras, can lead to similar multi-layered cell dysplasia [80]. In some cases, commensal bacteria, ISC tumors and the JNK pathway form a forward feedback loop that facilitates the tumor progression, JNK activation in ISC tumors causes them to secrete matrix metalloproteases, which disrupt the epithelial barrier and enable the overgrowth of commensal microbiota. This, in turn, fuels tumor progression and JNK activation [81].

Intestinal tumorigenesis in mammals can be driven by JNK activation [82]. The pathway has been implicated in inflammatory bowel disease (IBD), but this is controversial and debated [83,84]. Nevertheless, treatments targeting the JNK pathway ligand TNFα have been in use in the clinic for the past two decades and can induce and maintain remission of moderate-to-severe IBD (reviewed in [85]). Future work will be needed to determine whether the multiple roles of JNK in the *Drosophila* midgut epithelium are at work in the mammalian intestine.

## 7. Concluding Remarks

The recent wave of discoveries presented in this review, many of them within the last three years for fly testis and the last 10 years for the fly gut, render a new perspective of the JNK pathway in adult stem cells, both as a maintenance factor and as a trigger for regenerative responses. During homeostasis, JNK signaling can act as a self-renewal factor, ensuring that “stemness” is retained in the resident stem cell populations. During regeneration, JNK can induce a myriad of cellular responses in stem cells, in addition to its traditional role of inducing apoptosis in damaged cells. These other responses include increasing proliferation, switching the mode of cell division, reprogramming cells, and recycling cellular debris. Furthermore, JNK signaling plays critical roles during pathological situations and aging. Research aimed at translating discoveries made in fruit flies to mouse models may have important implications for human health.

In closing, many questions about JNK signaling still remain. For example, in the future, it will be important to understand why some responses require extrinsic (such as TNFα) vs. intrinsic factors for pathway activation and to elucidate potentially new factors in JNK activation. It will be also critical to improve our understanding of how the diversity of target genes and protein substrates results in differential cellular responses depending on the cell context and the type of stress.

## Data Availability

Not applicable.
